# From Mindless Masses to Small Groups: Conceptualizing Collective Behavior in Crowd Modeling

**DOI:** 10.1037/gpr0000032

**Published:** 2015-08-17

**Authors:** Anne Templeton, John Drury, Andrew Philippides

**Affiliations:** 1 School of Psychology, University of Sussex; 2 Department of Informatics, Centre for Computational Neuroscience and Robotics, University of Sussex

**Keywords:** crowds, computer simulations, modeling, social identity, intragroup processes

## Abstract

Computer simulations are increasingly used to monitor and predict behavior at large crowd events, such as mass gatherings, festivals and evacuations. We critically examine the crowd modeling literature and call for future simulations of crowd behavior to be based more closely on findings from current social psychological research. A systematic review was conducted on the crowd modeling literature (*N* = 140 articles) to identify the assumptions about crowd behavior that modelers use in their simulations. Articles were coded according to the way in which crowd structure was modeled. It was found that 2 broad types are used: mass approaches and small group approaches. However, neither the mass nor the small group approaches can accurately simulate the large collective behavior that has been found in extensive empirical research on crowd events. We argue that to model crowd behavior realistically, simulations must use methods which allow crowd members to identify with each other, as suggested by self-categorization theory.

Computer simulations are increasingly used to monitor and predict behavior at large crowd events, such as mass gatherings, festivals, and evacuations. Recent approaches to crowd modeling have proved effective in explaining patterns in aggregates of people together in the same place, such as pedestrians in a busy street (e.g., Helbing, Molnar, Farkas, & Bolay, 2001; Moussaïd, Helbing, & Theraulaz, 2011) and small group behavior within crowd flow (e.g., Köster, Seitz, Treml, Hartmann, & Klein, 2011; Moussaïd, Perozo, Garnier, Helbing, & Theraulaz, 2010; Singh et al., 2009). However, as yet, computer modelers have not created models which can adequately simulate certain key psychological features of large crowd behavior.

In a commentary on collective behavior, Turner (1987) argued that instead of treating crowds as individuals without any connections to one another, we need to explain the mental unity of real life crowds where the crowd behaves as one. As Reicher (1984) states, “the fascination of crowd psychology lies in the fact that it seeks to account for behavior that shows clear social coherence—in the sense of a large amount of people acting in the same manner—despite the lack of either pre-planning or any structured design” (p. 1). There are numerous real world examples of such collective behavior, for example football supporters performing a Mexican wave, protestors chanting together, or people coordinating their egress movements in emergencies. In each case, there is not only a physical crowd—an aggregate of individuals in the same location—but also a psychological crowd, that is, a shared psychological unity in those individuals and hence coordinated behavior (Reicher & Drury, 2011). Indeed, in some crowd events there may be more than one large psychological group which exists within a physical crowd. For example, in the case of a football match, the fans of each team make up two psychological crowds that behave differently from each other within one large physical crowd of people in the same stadium.

For a number of years, researchers modeling crowd behavior have recognized that to enhance the realism of simulations, and to better approximate collective behavior, greater granularity or psychological detail is required (for examples see Galea, 2006; Gerodimos, 2006). Thus some modelers have explicitly looked to the social sciences for both evidence and concepts for understanding collective behavior (e.g., Franca, Marietto, & Steinberger, 2009; Fridman & Kaminka, 2007; Helbing, Farkas, & Vicsek, 2002; Johnson & Feinberg, 1997). In different ways, these and other modelers have argued that more accurate simulations will require the inclusion of *groups* within a crowd (e.g., Aguirre, El-Tawil, Best, Gill, & Fedorov, 2011; Bruno, Tosin, Tricerri, & Venuti, 2011; Singh et al., 2009). However, this raises the question of what is meant by the concept of ‘group.’ In both psychology and computer science there are different understandings of what is meant by a ‘group.’ Some of these understandings may be better than others in helping to produce a more realistic simulation of behavior in a psychological crowd.

This article will critically examine existing crowd computer simulations by first outlining how understandings of group and collective behavior have developed within social psychology, before presenting a systematic review of the implicit and explicit assumptions in modelers’ treatment of ‘groups’ and ‘crowds’. On the basis of this review we will argue that crowd modelers will benefit from incorporating aspects of self-categorization theory (Turner, 1982; Turner, Hogg, Oakes, Reicher, & Wetherell, 1987) in to their models in order to create realistic simulations of collective behavior in line with findings from empirical psychological research.

## Toward an Understanding of Collective Behavior

In early understandings of collective behavior, crowds were treated as either a mass of people under one ‘group mind,’ or a mass of numerous unconnected individuals within the crowd. In ‘group mind’ accounts, crowds were understood as homogeneous entities where upon entering a crowd individuals lost both their individual ability to reason and their personality. Here every crowd member became indistinguishable from the others as they tended toward indiscriminate violence (Le Bon, 1960). Individualist accounts, such as Allport (1924), argued that the idea of the collective is a nominal fallacy; groups and crowds are merely aggregates of individuals. Any collectivity was seen to occur only through social facilitation, whereby the presence of others stimulated behavior that was already present in each individual. Later research demonstrated that neither group mind nor individualism could explain the social form of collective behavior; the mechanisms posited by Le Bon, Allport and others to explain collectively were inherently primitive, irrational, and mindless. For both positions, collective behavior tends to indiscriminate violence. However, extensive empirical research has shown that most crowds are not violent, and that even in riots and violent crowds, behavior is rational, discriminate, and often shows a pattern which is in line with shared conceptions of legitimacy (e.g., Fogelson, 1971; Reicher, 1984, 1996; Reicher & Stott, 2011; Thompson, 1971).

In the current literature, collective behavior is often characterized as ‘contagion’ where the mere sight or sound of others’ behavior apparently influences individuals in a crowd to behave in the same way (e.g., Gallup et al., 2012; Mann, Faria, Sumpter, & Krause, 2013). However, social psychologists examining crowd behavior have argued that the concept of ‘contagion’ cannot explain group boundaries to social influence. Thus Milgram and Toch (1969) pointed out that a different model of collective behavior was required to explain why the rousing effects of a demagogue affected the behavior of protesters but not the riot police who were physically copresent in the same crowd. Psychological group boundaries in ‘contagion’ have also been demonstrated experimentally (Van der Schalk et al., 2011).

Later interactionist approaches focused on group norms and interactions, and treated groups as psychological entities. Asch (1952) claimed that to understand the individual we must pay some attention to the group they belong to on the principle that the parts get their meaning from their relationship within the whole. Sherif (1967) proposed that being in a group has psychological consequences which are separate to those of the individual, and collectivity emerged when individuals had shared meanings and beliefs. The ideas of these and other Gestalt social psychologists were crucial for influencing psychological research to view individuals as members of a shared social field which was separate from them as individuals. Some sociologists began to take up this idea of interaction and applied it to crowds by focusing upon meaning-seeking and social norms for individuals to gauge acceptable behavior in a novel situation where how to behave is not immediately obvious (Emergent Norm Theory: Turner & Killian, 1957, 1987).

Other sociologists such as Aveni (1977) criticized previous research for treating crowds as “spatially proximate collections of individuals . . . undergoing some common experience” (p. 96) and also noted that previous research has paid little attention to the structure of crowds. Aveni’s criticism of this approach was followed by research looking at the affiliation between some members of the crowd. Various studies showed that in an evacuation people will attempt to remain with the small group that they have preexistent affiliative bonds with, such as friends and family, even if this results in their evacuation time increasing or causing a hazard to themselves (Johnson, 1988; Mawson, 2005; Sime, 1983). However, approaches to crowd behavior focusing on small groups fall short of explaining large collective behavior. For example, these accounts cannot explain why in emergency situations a crowd of strangers can become united and help those who were previously strangers (Drury, 2012), or even that two large psychological crowds can exist who act together (intragroup) yet oppose one another (intergroup) (Reicher, 1996). Although there are many theories of crowd behavior, such as the individualist and contagion approaches mentioned above, one of the most widely accepted and utilized accounts of collective behavior in social psychology, which is grounded in extensive empirical research and can explain the collective behavior of psychological crowds, is self-categorization theory (Turner, 1982; Turner et al., 1987).

## The Psychological Crowd: A Self-Categorization Approach

Self-categorization theory suggests that shared social identity—people’s cognitive representation of their relationship to others - is what makes collective behavior possible (Turner, 1985). Self-categorization theory can therefore explain how physical aggregates of individuals can come together psychologically within a crowd and how a single physical crowd may consist of one, two (or more) psychological crowds who each act as a large group without prior interpersonal relationships or interpersonal interaction. Self-categorization theory suggests that collective behavior occurs through the process of *depersonalisation*. Here, individuals self-stereotype themselves in line with the definition of a social category and see themselves as being interchangeable with others in their social category. In doing this, individuals shift from their personal identity to their identity as a member of a particular social group (Turner et al., 1987).

A plethora of crowd phenomena has been explained by self-categorization theory, such as urban riots (Reicher, 1984), mass emergency evacuations (Drury et al., 2009), religious mass gatherings (Alnabulsi & Drury, 2014), music festivals (Neville & Reicher, 2011), collective action (Drury, Reicher, & Stott, 2003), and ‘personal space’ in crowded locations (Novelli, Drury, & Reicher, 2010). An example of this behavior can be seen during the London bombings of July 7th 2005, where individual commuters became united through a shared identity in relation to the threat of the bombs. On the basis of their shared identity, the commuters helped each other and reported feelings of ‘unity,’ and felt ‘part of a group’ (Drury, Cocking, & Reicher, 2009, p. 81). The ability of self-categorization theory to explain behavior in numerous situations indicates that modelers would benefit from applying this theory to their models in order to adequately simulate a broader variety of crowd behavior.

Over the past decade, there has been an increased recognition among modelers that the concept of social identity is necessary for more realistic crowd simulations (for examples, see Aguirre et al., 2011; Köster et al., 2011; Langston, Masling, & Asmar, 2006; Smith et al., 2009). Here we examine whether any computer models of crowds have responded to this perceived need and adequately implemented a model of crowd behavior in line with empirical research in crowd psychology. The following section will address the main modeling techniques which have been used to simulate crowds before we present the analysis of the conceptions of crowd behavior found in the modeling literature.

## Psychological Requirements for Modeling the Crowd

Social psychological research on crowd psychology suggests a set of theoretical criteria that computer simulations of crowds should adhere to. In particular, a simulation must be able to model individuals who have the required perceptual and cognitive abilities to recognize identities—both their own and others’. Modeling techniques such as flow-based models which treat all members of the crowd as identical (e.g., Fang, Lo, & Lu, 2003) are inappropriate as they cannot model the variable cognitive processes in individuals. Two commonly used approaches for simulating crowd behavior are social force models and cellular automata. Both model types are typically based upon set rules and equations which have the same rules for every individual. In these models, the behavior of individuals is determined by attractors and repellors such as attraction to an area in the virtual environment and repulsion from other individuals to avoid collision (e.g., Burstedde, Klauck, Schadschneider, & Zittartz, 2001; Zhao, Yang, & Li, 2008; Zhang, Zhao, & Liu, 2009).

However, other models such as agent-based models (ABMs) do have the potential to simulate these individual capacities as each agent can have different characteristics which affect their behavior. ABMs can represent varying levels of perceptual and cognitive processes. Importantly, they are also dynamic, as the behavior of the agents (people) within the crowd, their individual characteristics, and the ‘information’ that the agents receive, together drive their actions and can be updated at each time step of the simulation (e.g., Fang, Yuan, Wang, & Lo, 2008; Ji & Gao, 2007; Köster et al., 2011). ABMs thus lend themselves to modeling complex crowd behavior and, in particular, situations in which individuals’ characteristics alter as their social identities change during the simulation. They can also represent more complex abilities, specifically the ability of individuals to perceive their own group membership and the group membership of other agents in the simulation. For instance, membership has been used to alter agent behavior through governing an agent’s spatial location based on the perception of their own group membership and the group membership of others, such as in leader and follower models (e.g., Qiu & Hu, 2010; Yuan & Tan, 2007). As such, ABMS have the ability to simulate psychological components of group identity and self-categorization in crowds. In this review, we will explore how the principles of identity and categorization have been implemented in existing ABMs and similar models of crowd behavior.

## Method

### Reviewing the Literature

**Figure 1 fig1:**
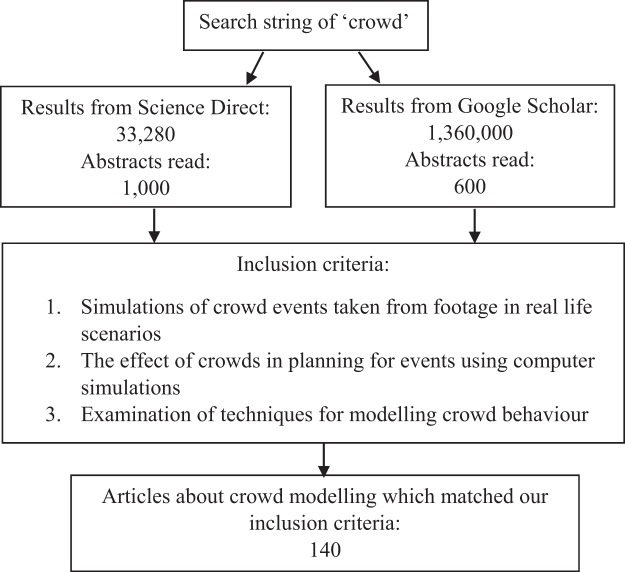
Flow diagram of search criteria and exclusion process for relevant articles. Where the same articles were generated by both Science Direct and Google Scholar, the abstracts were read and incorporated into the corpus only once.

A systematic review of the crowd modeling literature was conducted, in which publications were coded according to the psychological basis used to model crowd behavior. Literature was sourced from the ScienceDirect database and Google Scholar search engine (see Figure 1). In order to locate the relevant literature, the search string of “crowd” was used. Articles and conference proceedings about crowd models were selected from the generated results. Publications recommended by ScienceDirect due to their similarity to the articles identified were also incorporated in to the collection, and the references cited in relevant literature were also used to source additional literature.[Fig-anchor fig1]

### Crowd Modeling Typology

Each article was analyzed according to how the behavior of the crowd was treated. Where the theoretical basis for the crowd behavior being implemented was not explicitly stated by the authors, it was inferred from how the crowd behavior was modeled and what psychological literature was referenced, if any. Throughout data collection, it became evident that in the literature the crowd was conceptualized and implemented in one of five possible subtypes. These subtypes fit in to two major types. In the first type, the crowd is treated as a *mass*. In the second type, the crowd is treated as consisting of a number of *small groups*.

The prima facie validity of the subtypes was established by presenting descriptions of each category (with examples) to an audience of crowd modelers. To ascertain that the reliability of the subtypes by the first coder were correct, an interrater reliability analysis was conducted on the scheme used to divide cases into types and subtypes. Fourteen articles were randomly selected, and for each article an excerpt was chosen which represented the approach taken toward crowd behavior (minimum length of excerpt = 107 words, maximum length of excerpt = 341). These excerpts were presented to an independent judge, along with definitions of each subtype, and she assigned each article to a subtype. There was very good agreement between the allocation of the raters, Cohen’s Kappa K = .898 (*p* < .001) 95% CI (0.676, 1.000).

## Results

**Figure 2 fig2:**
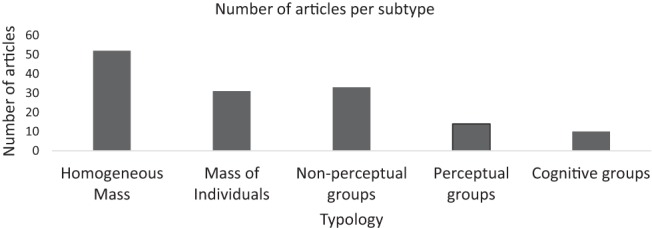
The number of articles published in journals and conference proceedings per subtype.

The most prominent models were the *mass* approaches to crowd behavior, which could be divided in to two subtypes; the ‘*homogeneous mass’* approach (52 articles) and the ‘*mass of individuals’* approach (31 articles). Within the *small groups* type, small groups are included in the crowd simulations but the understanding of ‘groups’ and methods to implement group behavior varied. Thus there were three subtypes of small group simulations; ‘*non-perceptual groups*’ (33 articles), ‘*perceptual groups*’ (14 articles), ‘*cognitive groups*’ (10 articles). The number of articles in each subtype is shown in Figure 2, and the allocation of all models in to subtypes is shown in Table 1.[Fig-anchor fig2][Table-anchor tbl1]

### Mass Crowd Simulations

Simulations which fall in to this category treat crowds as consisting of numerous ‘individuals’ in a large mass. Despite research demonstrating that there are often small psychological groups within physical crowds and extensive research showing that collective behavior requires individuals to see themselves as part of a large psychological crowd or group, groups are not implemented in these types of models.

### ‘Homogeneous Mass’ Subtype

The most commonly used approach within the crowd modeling literature is the *homogeneous mass* subtype. In examples of this subtype, the crowd is treated as an aggregate mass where every person is allocated identical properties. Within this subtype the crowd is regarded as a very large physical mass of individuals who coincidentally share the same goal—for example evacuating their environment. Literature in this subtype is therefore also characterized by modeling very basic agent behavior, often simply avoiding collisions with one another. This approach is predominantly used in order to model the effect of crowd size and crowd density on egress in emergency evacuations and ordinary environments. For example, Fang, Lo, and Lu (2003) modeled a crowd flow pattern in an emergency situation to examine the effect of crowd density on the speed of evacuation. Similarly, to examine the effect of crowd size on the speed of egress, Lee and Hughes (2006) manipulated the size of the crowd and the complexity of the environment to determine the effect on pedestrian walking speed. Although the assumptions underlying this approach are adequate to model the movement of one psychological crowd in a specific situation, these assumptions cannot accurately capture the behavior of crowds in more complex scenarios, such as when there are two or more crowds acting in different ways or even in contraflow. When other crowds are introduced in to the model, modelers need to simulate different crowd movement and dynamic group identities. Thus, the assumptions of this subtype cannot be applied to other scenarios where there is more than one psychological crowd.

### ‘Mass of Individuals’ Subtype

The *mass of individuals* approach differs from the homogeneous mass approach in that agents are given unique properties which make them act as individuals within the crowd. Usually, individual differences are implemented in order to examine the factors that can affect evacuation egress. For example, Shi et al. (2012) assign individuals different attributes such as response time, walking speed, and endurance in order to create a more realistic simulation of pedestrian evacuation in a heterogeneous crowd in a metro station in China. Other example attributes include different pedestrian velocities or health status (e.g., Dou et al., 2014). As in the previous subtype, the crowd members act independently but with the same goal of evacuating as quickly as possible. Some models include elaborate environments which affect the egress of individuals in more realistic simulations; for example Shi, Ren, and Chen (2009) manipulate the egress time of individual agents by causing the agents to be affected by the level of smoke in the room and how injured the individuals are. However, although these models can become very intricate, the premise of the model is still that of individual behavioral differences within a ‘mass’, rather than acting as a collective.

### Small Group Types

This subtype is characterized by small groups within the crowd. The small groups are usually implemented to determine the effect of groups on egress time, following Aveni’s (1977) research that suggested that crowds may be comprised of small groups and individuals. The type of groups that are implemented varied and can be divided in to three subtypes on an ordinal scale of psychological realism. However, all of these models represent sociality merely in terms of relations within small groups where collective behavior is reduced to being similar to interpersonal behavior rather than the crowd being a group itself.

### ‘Non-Perceptual Groups’ Subtype

Models of this subtype simulate physical groups but not psychological groups. That is, groups are implemented as homogeneous physical aggregates of people with no intragroup connection or individual knowledge of group membership. Instead, these are essentially small preexisting groups, which physically stick together in the crowd regardless of the situation. Thus, they move as one homogeneous aggregate, as though they are one large and slow individual. Simulations which fell in to this subtype model small groups in order to investigate the effect of groups on egress, particularly at bottlenecks and exits (e.g., Idrees, Warner, & Shah, 2014).

The implementation of small groups in this type of simulation is in some ways similar to the ‘mass of individuals’ approach. Instead of being an individual who acts independently within the mass, the group is an aggregate cluster of individuals which act as one within the crowd. Although no psychological connection between the groups is modeled, affiliative theories are often referenced (e.g., Aveni, 1977) to justify the inclusion of a group which stays together in a crowd situation (e.g., Feinberg & Johnson, 1995). For example, Dogbe (2012) modeled group behavior using attraction and repulsion interactions, where social groups (assumed to be friends and family in this model) are attracted to move together throughout the simulation, but are repulsed by other neighboring groups. By implementing group behavior in this way, Dogbe is simulating a crowd where the groups are essentially small numbers of people clumped together within the crowd, with no meaningful interaction other than to change formation in order to stay together as they move throughout the crowd. Although it is an advance in terms of psychological theory used that these models simulate groups which are visible through their movement, the focus on small groups neglects the fact that groups can coincide and that an entire crowd can move together as a unit.

### ‘Perceptual Groups’ Subtype

In contrast to the non-perceptual groups subtype, in perceptual groups individuals are able to perceive their own group membership, the identity of others within the crowd, and act according to their role. Often models which fall in to this subtype include ‘leaders’ and ‘followers’ where followers are treated as being together as a group because of their connection to leaders as the simulation unfolds (e.g., Moore, Flajšlik, Rosin, & Marshall, 2008). Although in simulations of this subtype, individuals are able to perceive their own group membership and the group identity of other individuals, their movement is derived from the idea that people will come together as a group because they are looking for signs and information about how to act in a novel situation. This approach to group behavior draws close parallels with emergent norm theory (Turner & Killian, 1957, 1987), as the agents are in a novel situation and look for leaders and social norms to discern how to act. However, a common problem with these models is that the agent’s priority is to move to the nearest leader, which causes clusters of individuals to form groups without the individuals ever having a psychological bond with any other person (e.g., Qui & Hu, 2010). This could be criticized as these groups are based upon being in the same spatial location rather than being together because they share a group identity, and agents have no perception of others aside from avoiding collision and knowing who is a ‘leader’ or a ‘follower.’

### ‘Cognitive Groups’ Subtype

In this subtype, individuals are able to perceive their own group membership and the group membership of others, just as in the ‘perceptual’ models. However, there is an extra component; individuals can share similar properties which are treated as ‘cognition’ by the authors. Here, agents who share the same properties are treated as being in a group. Additionally, the properties of each agent can change throughout the simulation, which causes the groups to change. As new information about the environment is given to the agents, the agents adapt their properties and seek out who they perceive to match their properties. Within this subtype, articles again tend to reference emergent norm theory (Turner & Killian, 1957, 1987) to justify why they implement interaction between crowd members. For example, Franca, Marietto, and Steinberger (2009) assign each agent certain properties. When new information is introduced to the agents, the agents begin to communicate to establish new norms. They seek out others who share their properties or are affected in the same way by information, and consequently move into groups with agents who share the same properties as them.

The principles behind simulations of the ‘cognitive’ subtype are the closest to psychological realism and lend themselves to more diverse implementations of both group and individual behavior. This approach is closest to self-categorization theory because it allows for the implementation of both individual properties and the ability to become a group member. It has also been used to simulate people acknowledging their group membership but being able to decide whether to act with their group or to act as an individual. Yuan and Tan (2007) created a scenario where a crowd of people have to evacuate a room, but agents can decide whether to leave with their group members or not. Moreover, this subtype focuses on the fact that groups exist based on shared properties, which is theoretically in line with the proposal of self-categorization theory that groups exist due to a sense of commonality between their members.

### Trend Analysis

**Figure 3 fig3:**
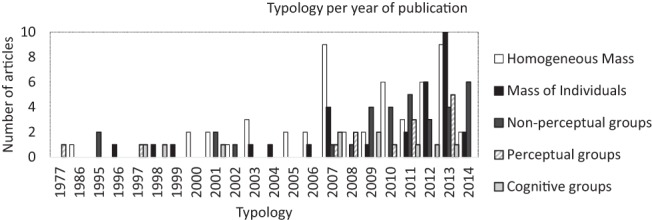
Prevalence of subtype per year of publication.

As Figure 3 shows, although the initial models of crowd behavior began with a mix of articles from all subtypes, since 2007 the ‘*homogenous mass*’, ‘*mass of individuals*’ and ‘*non-perceptual*’ subtypes have been more prominent. Although there was an initial spike of articles in the ‘*cognitive groups’* subtype in 2001, then another in 2009, this subtype has largely been overtaken by the ‘mass’ approaches. One factor which could have contributed to the rise in crowd modeling articles over the years is increased access to crowd modeling software. The upsurge of crowd simulations—particularly in the ‘homogeneous mass’, ‘mass of individuals’ and ‘non-perceptual groups’ subtypes - over the last decade could be due to the availability of modeling software such as SIMULEX (e.g., see Thompson & Marchant, 1995) and FIREScape (e.g., see Feinberg & Johnson, 1995), which provide tools to simulate crowds without focusing on group behavior (for a detailed analysis of emergency evacuation simulation models, see Santos & Aguirre, 2004).[Fig-anchor fig3]

## Discussion

### Misrepresenting the Crowd

This review has discerned that a plethora of models of crowd behavior have successfully simulated crowds of individuals. Notably, the majority of models have not aimed to incorporate psychological theories in to their rationale for crowd behavior. However, to accurately monitor and predict the collective behavior exhibited in psychological crowds specifically, it is imperative that models being used for crowd safety management have an accurate understanding of collective behavior taken from empirical research. In line with what is known in crowd psychology, a realistic model of collective behavior must include the capacity to simulate the difference between physical crowds and psychological crowds. Specifically, it must be able to model both the members of a crowd categorising themselves as individuals distinct from other individuals, and the situation where the same individuals categorise themselves as members of the crowd and hence share an identity. Simulations of psychological crowds must therefore address the way in which people can identify with one another and how collective behavior emerges from this process.

This review has found that some crowd modelers have begun to approach psychological realism by incorporating groupness (e.g., Aguirre et al., 2011; Moore, Flajšlik, Rosin, & Marshall, 2008) in their models of crowd behavior, particularly those we denoted as the ‘*cognitive groups’* subtype. However, these developments have not occurred at the same rate. Over the previous decade, there has been an increase in models which have implemented the ‘homogeneous mass’, ‘mass of individuals’ and ‘non-perceptual group’ approaches. The advantages and limitations of each subtype will be discussed, and we propose the theoretical advances that must be made in order for crowd models to simulate collective behavior more accurately across a variety of collective behavior scenarios.

### Constructing the Relationship Between the Individual and the Group

Examples of the mass type of model support Sime’s (1985) assertion that in computer simulations people are treated as ball-bearings; they are unthinking and act at a very base level of simply moving without interacting with one another. The *homogeneous mass* approach is also similar to the Le Bonian (1895) notion of crowds as an unthinking mass who act at a primitive psychological level, where there is no sense of individuality and thus is reminiscent of the broader mass society narrative, where the crowd is treated as an ‘undifferentiated whole’ (Giner, 1976, p. 47); the mass lacks capacity for moral sense, or a sense of direction. Models in this subtype are not behaviorally realistic because there are no individuals, and therefore there is no room for individual cognition from which meaningful group behavior can emerge. As mentioned previously, although models in this approach can simulate one crowd where members move together in a limited number of scenarios such as evacuation through one route, this account cannot explain collective behavior in all situations, such as where there are two or more psychological crowds, or two crowds in contraflow.

In Galea, Owen, and Lawrence’s (1996) model, the importance of each member of the crowd having individual attributes which change how people act throughout the simulation is emphasized. Although this was an important development for approaching psychological realism, it was at the cost of modeling collective behavior. Granularity is obtained at the cost of collectivity. In the mass of individuals approach, there is no collective behavior because the crowd members act as individuals without any sense of the commonality which is required for collective group behavior. To create a realistic model of collective behavior, modelers need to understand how the individual can become part of a psychological crowd. Thus, modelers need to implement the capacity of crowd members to act either as an individual or as a member of the crowd depending upon whether the person categorises themselves as an individual within a physical crowd, or as a member of the psychological crowd.

### The Crowd as Small Groups

Unlike the ‘*mass*’ type, models within the ‘*small groups*’ type have various levels of connections between the members of the crowd. The models in this subtype are a significant development in crowd modeling as they recognize and implement the importance of groupness and how this can affect the behavior of the crowd members. However, the ‘small groups’ type falls short of realistically modeling large crowd behavior as it only includes small groups within a crowd. Increasing granularity (small-group level variation within a physical crowd) loses the sense of ‘groupness’ at the crowd level because the focus is upon numerous small groups within the crowd. The approach therefore does not explain *collective* behavior where the crowd is one group. By doing this, the models are unable to simulate the behavior of large psychological crowds where the entire crowd shares one group identity. However, each subtype within the ‘small groups’ type has its own specific advantages and drawbacks.

In the ‘non-perceptual groups’ subtype of simulation, groups are treated as physical entities rather than being together due to a psychological bond between the members of the group. The original models in this subtype (e.g., Goldenstein et al., 2001; Thompson & Marchant, 1995) were very important for the development of simulations of crowd behavior because they introduced groups in to the crowd. However, groups are only incorporated in order to make simulations more realistic by claiming that the groups are families or friends. Group membership has no effect on the behavior of the group apart from staying together throughout the simulation. Although there are now groups, there is no sense of collective behavior based on a shared group identity.

Within the ‘perceptual groups’ subtype, modelers represent crowd members as being able to know their own group identity and the group identity of others. Although the ability of the crowd members to perceive group membership and act in accordance with it is in line with self-categorization theory (Turner et al., 1987), here groups are treated simply as people that are in the same spatial location. Although group membership is dependent upon group members actively categorising themselves as members of that specific group, group membership is limited and only goes as far as crowd members having roles as either a ‘leader’ or a ‘follower’ as opposed to group membership arising from a sense of common identity. Empirical research on group behavior suggests that psychological group membership is more versatile than this; when people are in a novel crowd situation they can come together through sharing a group identity and act together in a coordinated way, such as by self-organizing and helping one another (Drury, 2012; Drury et al., 2009). In addition, group membership does not need to be limited to those people within the same spatial location. The shared group identity can spread to include the entire crowd, where people have a shared social identity with others in the crowd and act in a coordinated way with them even if they are not near to each other, for example football fans in a stadium.

### Incorporating Cognition for Collective Behavior

The subtype of model which comes closest to explicating the underlying components of cognitive group membership and which is consistent with psychological research is the ‘cognitive groups’ subtype. Examples of this subtype not only incorporated the perception of group membership, but also went further than the ‘perceptual groups’ subtype by incorporating what is claimed as ‘cognition.’ In this subtype ‘cognition’ is instantiated as the ability of people to perceive their own beliefs and the beliefs of others, and group membership is dependent upon shared beliefs and desired actions. Moreover, in some simulations (e.g., Yuan & Tan, 2007), the agents are able to choose whether to act with a group or to act as an individual.

The incorporation of ‘cognition’ brings this subtype closest to implementing principles of self-categorization theory in a crowd simulation. Although not explicitly stated in any of the literature that has been reviewed, it could be argued that models in this subtype actually model something of the cognitive shift from being psychologically an individual to becoming a member of a particular social group and taking on that salient identity, which is crucial for collective behavior to emerge. However, despite these advantages, this approach does not completely model a psychological crowd as the models are yet to make the leap from small ‘cognitive’ groups to large crowds where the members share the same group identity. For example - although not specifically a model of crowd behavior - van Rooy (2012) uses an ABM to examine SCT by grouping individuals depending upon their shared opinions. Within this model the individuals could communicate their opinions with others and change group affiliation to be with others who shared the same opinions. By defining groups as those who share common opinions van Rooy’s definition of groups approaches psychological realism by basing group membership on a sense of commonality. While groups are still treated as consisting of small numbers, future work could ascertain whether these principles could be extended to an entire crowd.

### Toward a Cognitive Model of Collective Behavior

There are a number of factors that must be addressed in order for modelers to create an accurate simulation of collective behavior. One component that is fundamental to collective behavior is the perception of groupness: the ability of an individual to know their own group identity and perceive the group identities of others. An issue here is how to quantify the level of identification that a member feels with their group. Identification with a group is not simply a binary ‘identify’ or ‘do not identify’ scenario; modelers should create agents with the potential for variable levels of group identification which are dependent upon the context that the individual is in. Similarly, the effect of group identity upon behavior is not necessarily linear. Although an increased level of identification may cause individuals to behave in line with the norms of the group, other variables may act as moderators, such as beliefs about legitimacy of actions and levels of self-efficacy. One example of a model which has effectively employed aspects of self-categorization theory to simulate collective crowd behavior is von Sivers, Templeton, Köster, Drury, and Philippides (2014). The study described in this article is a first step toward examining the effect of self-categorization theory upon collective crowd behavior during an emergency, and could be used as a marker for future work simulating collective behavior.

This has been the first comprehensive and up-to-date review of how computer models have conceptualized groups and crowd behavior. Despite the importance that models used for crowd management and safety are able to realistically simulate crowd behavior, until now there has not been a review of how modelers approach collective behavior, or indeed whether they approach it at all. An earlier review by Sime (1985) found that the idea of ‘mass panic’ was influential in how modelers implemented crowd behavior in safety planning and the design of public spaces. However, modeling approaches have evolved since Sime’s review. There has been an upsurge in the number of crowd simulations since then, with some articles even referencing Sime in their justification for their new approaches to modeling crowd behavior (e.g., Feinberg & Johnson, 1995; Kamkarian & Hexmoor, 2013). In addition, a recent review of building evacuation simulations by Aguirre et al. (2011) found that modelers using ABMs placed an emphasis on individuality and mass panic and suggested that evacuation simulations need to include other social scientific factors such as norms, leadership, and group identification and membership.

Though both of these reviews were very important for addressing improvements the needed to be made in the crowd modeling literature, our review has gone further than this. We have comprehensively reviewed a broad scope of crowd modeling scenarios from 1977 to 2014, including simulations of crowd events taken from real life events, simulations of crowds in planning for events, literature looking at techniques for modeling crowd behavior using simulations, and articles which addressed the techniques used to model crowd behavior. Moreover, we have examined the theoretical underpinnings of each of these 140 articles to determine what assumptions modelers are making about crowd behavior. This is the first systematic comparison of the crowd modeling literature with current models of crowd behavior in social psychology.

By examining what crowd modelers are creating and comparing it to empirical research of collective behavior, we can see what future models need to change. Although models have been successful in simulating crowds without a group identity, as yet simulations have not aimed to model large psychological behavior. Modelers are yet to model the transformation of people from identifying as an individual to identifying as a member of the crowd. Without this they cannot model meaningful collective behavior where the behavior of a large crowd can be understood in terms of group membership, which is needed to explain scenarios where there is more than one crowd present (such as the football fans mentioned previously). To create a realistic model of crowd behavior, crowd modelers must look to the extensive empirical research on group and crowd behavior in social psychology.

We propose that to make more realistic simulations of collective behavior, which can be applied to a broad range of scenarios, modelers must implement aspects from self-categorization theory. Specifically, these simulations should be based on the aspects of self-categorization theory which can explain how members of a large crowd share the same group identity, the transformation from the individual identities to the identities as group members, and the subsequent actions which follow from being part of that group. While this would create more realistic models of collective behavior for modelers, this interdisciplinary work could also benefit social psychologists. By creating models which incorporate self-categorization theory and accurately simulate the behavior that we have found in empirical search, it could help to develop theories of collective behavior in social psychology. Only by incorporating these aspects that are based on extensive empirical social psychological research will crowd modelers be able to realistically simulate, monitor, and predict collective behavior in crowds across a wide range of crowd events.

## Figures and Tables

**Table 1 tbl1:** List of the Literature Reviewed and the Allocated Subtypes

Authors	Year	Typology
Aguirre, El-Tawil, Best, Gill, & Federov	2011	Perceptual groups
Andrade, Blunsden, & Fisher	2006	Homogeneous mass
Banarjee, Grosan, & Abraham	2005	Homogeneous mass
Bandini, Gorrini, & Vizzari	2014	Perceptual groups
Bicho, Rodrigues, Musse, Jung, Paravisi, & Magalhaes	2012	Non-perceptual groups
Bierlaire, Antonini, & Weber	2003	Mass of individuals
Bodgi, Erlicher, & Argoul	2007	Homogeneous mass
Bruno, Tosin, Tricerri, & Venuti	2011	Homogeneous mass
Burstedde, Klauck, Schadschneider, & Zittarz	2001	Homogeneous mass
Carroll, Owen, & Hussein	2013	Homogeneous mass
Chen & Huang	2011	Non-perceptual groups
Chen & Lin	2009	Non-perceptual groups
Chen, Wang, Wu, Chen, Khan, Kolodziej, Tian, Huang, & Liu	2013	Perceptual groups
Cho & Kang	2014	Non-perceptual groups
Chong, Liu, Huang, & Badler	2014	Non-perceptual groups
Chow	2007	Homogeneous mass
Chrysostomou, Sirakoulis, & Gasteratos	2014	Non-perceptual groups
Davidich & Köster	2013	Mass of Individuals
Degond & Hua	2013	Homogeneous mass
Dogbe	2012	Non-perceptual groups
Dou, Chen, Chen, Chen, Deng, Zhang, Xi, & Wang	2014	Mass of Individuals
Fang, Lo, & Lu	2003	Homogeneous mass
Fang, Yuan, Wang, & Lo	2008	Homogeneous mass
Fienberg & Johnson	1995	Non-perceptual groups
Franca, Marietto, & Steinberger	2009	Cognitive groups
Fridman & Kaminka	2007	Non-perceptual groups
Galea, Owen, & Lawrence	1996	Mass of individuals
Gawroński & Kulakowski	2011	Perceptual groups
Georgoudas, Kyriakos, Sirakoulis, & Andreadis	2010	Homogeneous mass
Goldenstein, Karavelas, Metaxas, Guibas, Aaron, & Goaswami	2001	Non-perceptual groups
Gutierrez, Frischer, Cerezo, Gomez, & Seron	2007	Mass of individuals
Haciomeroglu, Barut, Ozcan, & Sever	2013	Non-perceptual groups
Heïgeas, Luciani, Thollot, & Castagne	2003	Homogeneous mass
Helbing, Johansson, & Al-Abideen	2007	Mass of Individuals
Helbing, Farkas, & Vicsek	2000	Homogeneous mass
Helbing, Farkas, Molnar, & Vicsek	2002	Homogeneous mass
Helbing, Molnar, Farkar, & Bolay	2001	Non-perceptual groups
Heliövaara, Korhonen, Hostikka, & Ehtamo	2012	Homogeneous mass
Henein & White	2007	Homogeneous mass
Hu, Zheng, Wang, & Li	2013	Mass of individuals
Hughes	2000	Homogeneous mass
Hussain, Yatim, Hussain, & Yan	2011	Non-perceptual groups
Idrees, Warner, & Shah	2014	Non-perceptual groups
Ji & Gao	2007	Perceptual groups
Ji, Zhou, & Ran	2013	Homogeneous mass
Jiang, Xu, Mao, Li, Xia, & Wang	2010	Non-perceptual groups
Jiang, Zhang, Wong, & Liu	2010	Homogeneous mass
Ji-hua, Cheng-zhi, Zhi-Feng, & Bo	2013	Homogeneous mass
Johansson, Batty, Hayashi, Bar, Marcozzi, & Memish	2012	Mass of individuals
Johnson & Feinberg	1977	Perceptual groups
Johnson & Feinberg	1997	Perceptual groups
Johnson, Hart, & Hui	1999	Mass of individuals
Kamkarian & Hexmoor	2013	Mass of individuals
Karni & Schmeidler	1986	Homogeneous mass
Khaleghi, Xu, Wang, Li, Lobos, Liu, & Son	2013	Homogeneous mass
Kirchner & Schadschneider	2002	Non-perceptual groups
Kirchner, Klüpfel, Nishinari, Schadschneider, & Schreckenberg	2003	Homogeneous mass
Köster, Seitz, Treml, Hartmann, & Klein	2011	Perceptual groups
Kountouriotis, Thomopoulos, & Papelis	2014	Perceptual groups
Krausz & Bauckhage	2012	Homogeneous mass
Lachapelle & Wolfram	2011	Non-perceptual groups
Langston, Masling, & Asmar	2006	Mass of individuals
Lee & Hughes	2006	Homogeneous mass
Lee & Hughes	2007	Homogeneous mass
Lei, Li, Gao, Hao, & Deng	2012	Mass of individuals
Li & Qin	2012	Homogeneous mass
Lister & Day	2012	Homogeneous mass
Lo, Fang, Lin, & Zhi	2004	Mass of individuals
Löhner	2010	Non-perceptual groups
Lozano, Morillo, Orduña, Cavero, & Vigueras	2009	Non-perceptual groups
Ma, Lo, Song, Wang, Zhang, & Liao	2013	Homogeneous mass
Ma & Song	2013	Perceptual groups
Manfredi, Vezzani, Calderara, & Cucchiara	2014	Non-perceptual groups
Marana, Velastin, Costa, & Lotufo	1998	Mass of individuals
Maury, Roudneff-Chupin, & Santambrogio	2010	Homogeneous mass
Mazzon, Tahir, & Cavallaro	2012	Mass of individuals
Mehran, Oyama, & Shah	2009	Non-perceptual groups
Mekni	2013	Cognitive groups
Moore, Flajšlik, Rosin, & Marshall	2008	Perceptual groups
Moussaïd, Helbing, & Theraulaz	2011	Mass of individuals
Moussaïd, Perozo, Garnier, Helbing, & Theraulaz	2010	Non-perceptual groups
Mukovskiy, Slotine, & Giese	2013	Homogeneous mass
Musse & Thalmann	1997	Cognitive groups
Musse & Thalmann	2001	Cognitive groups
Musse, Babski, Çapın, & Thalmann	1998	Cognitive groups
Narain, Golas, Curtis, & Lin	2009	Homogeneous mass
Nilsson & Johansson	2009	Non-perceptual groups
Oğuz, Akaydın, Yılmaz, & Güdükbay	2010	Non-perceptual groups
Pan, Han, Dauber, & Law	2007	Cognitive groups
Parunak, Brooks, Brueckner, & Gupta	2012	Cognitive groups
Pelechano, Allbeck, & Badler	2007	Mass of Individuals
Pires	2005	Homogeneous mass
Qui & Hu	2010	Perceptual groups
Ramesh, Shanmughan, & Prabha	2014	Mass of individuals
Ran, Sun, & Gao	2014	Non-perceptual groups
Ryan, Denman, Fookes, & Sridharan	2014	Homogeneous mass
Sagun, Bouchlaghem, & Anumba	2011	Homogeneous mass
Sarmady, Haron, & Talib	2011	Homogeneous mass
Shao, Dong, & Tong	2013	Non-perceptual groups
Shendarkar, Vasudevan, Lee, & Son	2008	Perceptual groups
Shi, Ren, & Chen	2009	Mass of individuals
Shi, Zhong, Nong, He, Shi, & Feng	2012	Mass of individuals
Silverberg, Bierbaum, Sethna & Cohen	2013	Homogeneous mass
Singh, Arter, Dodd, Langston, Lester, & Drury	2009	Non-perceptual groups
Smith, James, Jones, Langston, Lester, & Drury	2009	Cognitive groups
Song, Gong, Cui, Fang, & Cao	2013	Mass of Individuals
Spieser & Davison	2009	Homogeneous mass
Tajima & Nagatani	2001	Homogeneous mass
Thiel-Clemen, Köster, & Sarstedt	2011	Non-perceptual groups
Thompson & Marchant	1995	Non-perceptual groups
Tong & Cheng	2013	Mass of individuals
Varas, Cornejo, Mainemer, Toledo, Rogan, Munoz, & Valdivia	2007	Homogeneous mass
Vasudevan & Son	2011	Cognitive groups
Vigueras, Lozano, Orduña, & Grimaldo	2010	Homogeneous mass
Wagner & Agrawal	2014	Homogeneous mass
Wang, Li, Khaleghi, Xu, Lobos, Vo, Lien, Liu, & Son	2013	Homogeneous mass
Wang, Zhang, Cai, Zhang, & Ma	2013	Mass of individuals
Wang, Zheng, & Cheng	2012	Mass of individuals
Weifeng & Hai	2011	Mass of individuals
Wu & Radke	2014	Mass of individuals
Xiaoping, Wei, & Chao	2010	Homogeneous mass
Xiong, Cheng, Wu, Chen, Ou, & Xu	2012	Non-perceptual groups
Xiong, Lees, Cai, Zhou, & Low	2010	Homogeneous mass
Yamamoto, Kokubo, & Nishiniari	2007	Homogeneous mass
Yan, Tong, Hui, & Zonghhi	2012	Mass of Individuals
Yaseen, Al-Habaibeh, Su, & Otham	2013	Non-perceptual groups
Yu & Johansson	2007	Homogeneous mass
Yuan & Tan	2007	Cognitive groups
Yücel, Zanlungo, Ikeda, Miyashita, & Hagita	2013	Perceptual groups
Zanlungo	2007	Mass of individuals
Zanlungo, Ikeda, & Kanda	2012	Homogeneous mass
Zawidzki, Chraibi, & Nishinari	2013	Mass of Individuals
Zhang, Liu, Liu, & Zhao	2007	Homogeneous mass
Zhang, Liu, Wu, & Zhao	2007	Homogeneous mass
Zhang, Weng, Yuan, & Chen	2013	Mass of individuals
Zhao, Wang, Huang, Cui, Qui, & Wang	2014	Mass of individuals
Zhao, Yang, & Li	2008	Homogeneous mass
Zheng & Cheng	2011	Non-perceptual groups
Zheng, Sun, & Zhong	2010	Homogeneous mass
Zheng, Zhao, Cheng, Chen, Liu, & Wang	2014	Non-perceptual groups
